# Transmission of *Toxoplasma gondii* Infection Due to Bone Marrow Transplantation: Validation by an Experimental Model

**DOI:** 10.3389/fmed.2019.00227

**Published:** 2019-10-15

**Authors:** Carolina Salomão Lopes, Tamires Lopes Silva, Julio Cesar Neves de Almeida, Lucas Vasconcelos Soares Costa, Tiago Wilson Patriarca Mineo, José Roberto Mineo

**Affiliations:** Laboratory of Immunoparasitology “Dr. Mario Endsfeldz Camargo”, Institute of Biomedical Sciences, Federal University of Uberlândia, Uberlândia, Brazil

**Keywords:** bone marrow, transplantation, infectious diseases, *Toxoplasma gondii*, toxoplasmosis, acute infection, reactivation

## Abstract

Toxoplasmosis is an opportunistic infectious disease and may present a fatal outcome for human bone marrow transplant (BMT) recipients, due to the rapid disease course in immunosuppressed individuals. Several reports about occurrence of toxoplasmosis after BMT have been published in the literature, but this disease has been associated mainly due to reactivation of latent infection rather than primary infection. Even though there are reports of acute toxoplasmosis in recipients who were seronegative for *T. gondii*, suggesting transmission of infection after BMT, the source of infection in those cases has not been clearly demonstrated, whether it is due to the transplantation procedure by itself or from environmental source. Thus, the present study aimed to observe if it could be possible to demonstrate the parasite‘s ability to infect bone marrow (BM) cells and cause toxoplasmosis, when using an experimental model. Our results showed that 11% of hematopoietic and 7.1% of nonhematopoietic lineages may become infected when using *in vitro* experiments. Also, *in vivo* experiments demonstrated that, when C57BL/6 mice were infected with RH-RFP or ME-49-GFP *T. gondii* strains, the BM cells may be infected at different time points of infection. The parasites were detected by both fluorescent microscopy and qPCR. Also, when those BM samples were collected and used for BMT, the transplanted animals presented high rates of mortality and 87.5% of them became seropositive for *T. gondii*. Taken together, our results clearly demonstrated that it is possible to acquire primary *T. gondii* infection from the donor cells after BMT. Therefore, we are emphasizing that, before transplantation, serological screening for *T. gondii* infection from both donors and recipients, in addition to DNA search for this parasite from donor bone marrow cells, are necessary procedures to avoid the risk of *T. gondii* infection for immunocompromised patients.

## Introduction

*Toxoplasma gondii*, a worldwide protozoan parasite, may cause opportunistic disease in immunodeficient individuals (1, 2). The prevalence of *T. gondii* infection in humans differs widely from region to region, ranging from 10 to 80% (1, 3, 4). The parasite transmission occurs by ingestion of raw or undercooked meat containing bradyzoites within tissue cysts from infected animals, as chicken, pig, sheep, and others, or by ingesting oocysts shed into the environment and contaminating soil or water, as well as by transplacentary transmission, or by solid organ transplantation (2, 3). The infection in immunocompetent humans usually remains asymptomatic in 80% of the patients, or may present only flu-like symptoms (1, 5).

The parasite persists lifelong in the infected hosts, establishing a latent chronic infection which is usually harmless. However, severe cases can occur in transplacental transmission, when a woman becomes primary infected during pregnancy, or in immunocompromised patients, when primary or reactivated infections may occur. Also, severe cases may occur during post-transplant immunosuppressive treatment protocols in patients who received solid organs or bone marrow transplantation (1, 5).

Bone marrow transplantation (BMT) has become a frequent procedure to treat malignant hematologic diseases or congenital bone marrow disorders (6) and several reports of post-BMT toxoplasmosis have demonstrated that the course of infection is usually rapid and present a poor prognosis, which may lead to fatal outcome (7, 8). The frequent manifestations of reactivated toxoplasmosis are *T. gondii* encephalitis, myocarditis, pneumonitis, hepatitis, and ocular toxoplasmosis (9, 10). Data reported from these cases reinforce the process of reactivation of a latent infection in seropositive patients instead of a primary infection. Even though there is an increasing number of reports suggesting a possible transmission of *T. gondii* parasite by BMT (11–13), it is unclear if the infection occurs due to a result of donor-transmitted infection, or reactivation of latent infection, or *de novo* infection.

Considering the importance to understand the possibility of *T. gondii* transmission through BMT due to donor-transmitted infection, the aim of the present work was to demonstrate if an experimental murine model could be appropriate to answer this question, by using animals under acute or chronic *T. gondii* infection as bone marrow donors.

## Materials and Methods

### Animals

C57BL/6J mice, 7–8 weeks of age, were bred and maintained at animal facilities of Federal University of Uberlândia. This study was approved by The Committee for Ethical Use of Experimental Animals of Federal University Uberlândia (CEUA-UFU Protocol # 109/16), according to the procedures established by the Universities Federation for Animal Welfare.

### Parasites Strains

Tachyzoites of *T. gondii* RH-RFP and ME-49-GFP strains were maintained by serial passages in HeLa cells (ATCC® CCL-2™, Manassas, VA, USA). *T. gondii* RH strain stably expressing tandem tomato red fluorescent protein (RH-RFP) under tubulin promoter was previously generated by Striepen et al. (14) and kindly provided by Professor Vern Carruthers. *T. gondii* ME49 strain expressing GFP-Luciferase (ME49_GFP-Luc) under the DHFR promoter was generated by Saeij et al. (15) and kindly provided by Professor Érica Martins Duarte. The parasites were stained by Trypan blue and counted with a Neubauer chamber to determine the percentage of viable parasites before use in the *in vitro* or *in vivo* experiments.

### *In vitro* Experimental Procedures

Bone marrow cells were isolated from C57BL/6J compact bones and were co-cultured with ME-49-GFP strain in RPMI media for 18 hours (MOI 1:5) in 5% CO_2_ at 37°C incubator. Cells were detached from culture dishes for FACS experiments, and stained fresh with APC-Cy™ 7 Rat Anti-Mouse CD45 (BD Pharmingen™ cn/557659, San Diego, CA, USA). The acquisition and analysis were processed using the FACSCANTO II, BD.

### *In vivo* Experimental Procedures

Mice (*n* = 15) were infected with 10^2^ tachyzoites of RH-RFP strain by intraperitoneal route (i.p.), and another group of mice (*n* = 25) was infected with 10^3^ tachyzoites of ME-49-GFP strain by the same route. RH-RFP infected animals were euthanized at 3, 5, and 7 days after infection (dpi) and animals infected with ME-49-GFP strain were euthanized at 3, 5, 7, 15-, and 30-dpi, being five animals euthanized for each time point. Bone marrow was harvested from each animal to perform isogenic transplantation in naïve animals and the parasite detection was monitored by microscopy analysis and qPCR. Bone marrow cells were flushed from tibias and femurs of C57BL/6J mice with RPMI medium using a 25-gauge needle. Cells were stained with Trypan blue and counted in a Neubauer chamber to determine the percentage of viable cells.

Isogenic bone marrow transplantation was performed in five mice for each time point. The animals were intraperitoneally inoculated with 10^6^ bone marrow cells. Animals were followed up during 30 days to determine the seroconversion and the mortality rates. The seroconversion was evaluated at day 10 and 30 after transplant by indirect ELISA, as previously described (16). The results were expressed in ELISA index (EI) and EI values >1.2 were considered positive. On day 30, transplanted animals were euthanized and the bone marrow from these animals was evaluated by microscopy analysis to verify the presence of parasites.

### Microscopy Analysis

Fresh samples from BM were dropped on the slides and observed using fluorescence microscope (EVOS FL Cell Imaging System—Thermo Fisher Scientific—US) to detect the fluorescence of parasite.

### Parasite Burden

The parasite burden was evaluated by qPCR. To quantify the *T. gondii* DNA in BM samples, the DNA was extracted from the pelleted cells with a commercial kit (Wizard Genomic DNA purification Kit, Promega Co., Madison, WI, USA). The PCR protocol was performed using the SYBR green system (GoTaq qPCR Mater Mix, Promega) and the following primers targeting the Tg529 sequence of *T. gondii* was used Forward GCTCCTCCAGCCGTCTTG, Reverse TCCTCACCCTCGCCTTCAT.

GAPDH gene was used as an internal control (forward primer: 5′-GGAGAAACCTGCCAAGTATGATG-3′ and reverse primer: 5′-CAGTGTAGCCCAAGATGCCC-3′). All samples were standardized for 200 ng of DNA and run in duplicate. The analysis was performed with a real-time PCR thermal cycler (StepOne Plus, Life, Thermo Scientific). The results were expressed as picogram of parasite DNA per microgram of total tissue DNA extracted.

### Statistical Analysis

One-way ANOVA followed by Tukey's test was used for evaluation of parasite burden. The survival curve was estimated using the Kaplan-Meier method. Statistical calculations were performed using GraphPad software (version 6.0, GraphPad, La Jolla, CA, USA).

## Results

### *In vitro T. gondii* Infection of Bone Marrow Cells

**Figure 1:**
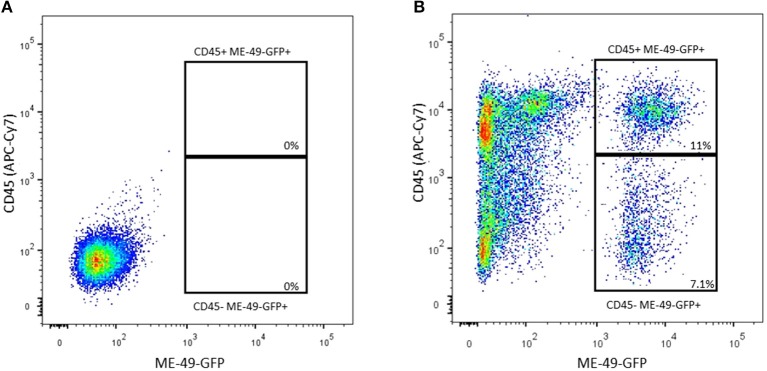
Flow cytometry analysis of BM cells co-cultured with ME-49-GFP *T. gondii* strain stained with anti-CD45 (APC-Cy7). **(A)** CD45(APC-Cy7)/ME49 (GFP) gate from control cells **(B)** CD45(APC-Cy7)/ME49 (GFP) gate from infected cells.

Bone marrow cells were infected *in vitro* by ME-49-GFP *T. gondii* strain, being subsequently stained by APC-CY7 anti-CD45 to verify the parasite capacity to infect hematopoietic (CD45+) and/or nonhematopoietic (CD45-) lineages. As shown in Figure 1, FACS analyses revealed that 11% of hematopoietic cells lineage turn out to be infected by ME-49-GFP, whereas 7.1% of nonhematopoietic lineage also became infected by the same parasite strain.

### Detection of *T. gondii* in Bone Marrow Samples From Donors' Mice

**Figure 2:**
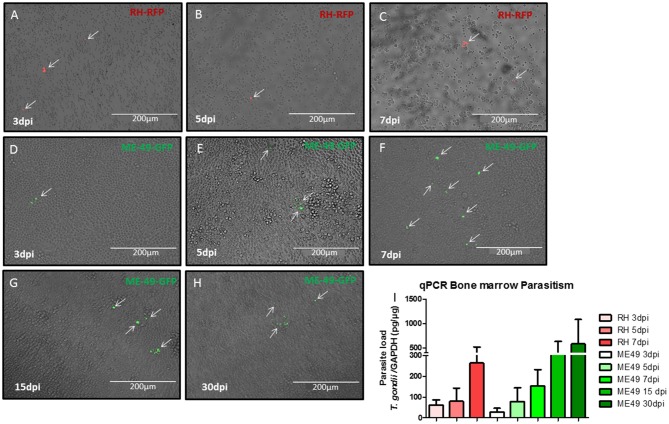
Assessment of parasite in bone marrow samples. Microscopic images of bone marrow cell from donors at different times of infection. Mice infected with RH-RFP at 3 days post infection **(A)**, 5 days post infection **(B)**, and 7 days post infection **(C)**. Mice infected with ME49-GFP at 3 days post infection **(D)**, 5 days post infection **(E)**, 7 days post infection **(F)**, 15 days post infection **(G)**, and 30 days post infection **(H)**. Arrows indicate the parasite presence. **(I)** Quantitative real-time PCR analysis of the *T. gondii* burden in BM samples from murine donors.

To evaluate the presence of *T. gondii* on BM during infection, cells from bone marrow were harvest from C57BL/6J mice in different times of infection to search for parasites. It was possible to observe the presence of intra and extracellular parasites in all samples analyzed (Figures 2A–H). Quantitative PCR were performed to detect parasite burden on BM samples and it was found an increasing parasitism along the time of infection (Figure 2I).

### Bone Marrow Transplantation

**Figure 3:**
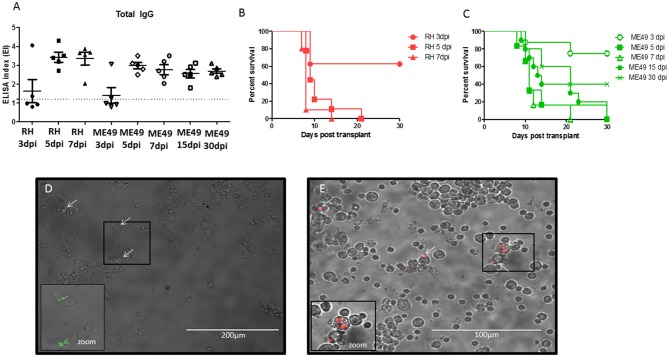
Evaluation of *T. gondii* infection in animals after bone marrow transplantation. **(A)** Levels of IgG antibodies anti-*Toxoplasma gondii* in serum samples of transplanted mice after 10 days of BMT. The values are expressed in ELISA index and dashed line indicates the cut off value = 1.2. **(B,C)** Survival curve of mice after BMT. **(D)** Representative image of bone marrow cells from recipient mice transplanted with BM from animals infected with ME-49-GFP after 30 days of BMT. **(E)** Representative image of bone marrow cells from recipient mice transplanted with BM from animals infected with RH-RFP after 21 days of BMT. Arrows indicate the parasite presence. Cropped image a zoom of cells infected.

Bone marrow samples from infected mice were transplanted via i.p. in isogenic recipients. The seroconversion was evaluated at day 10 and repeated at day 30 of infection to follow up the seroconversion. All animals transplanted at day 5, 7, 15, and 30 seroconverted after 10 days, whereas animals transplanted with BM collected at day 3 of infection had only 50% of seroconversion (Figure 3A). The seronegative animals had blood samples collected and tested at day 30, however no seroconversion was detected (data not shown). Thus, a total of 35 from 40 (87.5%) animals transplanted presented antibody against *T. gondii* by ELISA assay. The survival rates were also evaluated in animals transplanted with BM at different time points of infection, and they were monitored during 30 days after transplants. Animals that received BM from animals at 5 and 7 days of infection with RH-RFP or ME49-GFP died within 30 days (Figures 3B,C). Mice transplanted with BM samples from animals infected for 3 days presented a reduced mortality, corroborating with the results of seroconversion showing that there were animals from this condition that did not become IgG positive for *T. gondii*. In contrast, mice transplanted with BM from animals at 15 and 30 days of infection with ME49-GFP seroconverted and most of them succumbed due to infection along 30 days.

All surviving animals were euthanized at day 30 to evaluate the presence of parasite in bone marrow samples from recipients' mice. It was found parasite in all analyzed samples from animals seropositive for *T. gondii* (Figure 3D). Bone marrow from animals transplanted with samples from mice infected by RH-RFP at 5-dpi that died at day 21 was also examined. As shown in Figure 3E, it was also detected the presence of tachyzoites in this condition.

## Discussion

Bone marrow transplantation is a medical procedure applied mainly to the treatment of neoplastic diseases, as well as for other bone marrow disorders (17). The incidence of toxoplasmosis after this procedure ranges from 0.25% (18) to 12.9% (19), and appears to be unrelated to the regional seroprevalence of *T. gondii* infection (20). It has been described an increase of this disease after allo-BMT, which might present a fatal outcome, due to its rapid course, particularly in immunocompromised patients (12). Regarding the several cases reported in literature of toxoplasmosis after BMT, many investigators have concluded that it is the result of reactivation of latent infection rather than due to primary infection (21, 22). It is noteworthy to point out that, when analyzing several clinical cases reporting patients who were seronegative to *T. gondii* before transplant and acquired toxoplasmosis after the procedure, it is uncertain whether this protozoan disease occurred due to the bone marrow transplantation *per se* or by another source. Indeed, concerning the reports in the literature suggesting the transmission of *T. gondii* after BMT, it is not clear if it is caused by the transplantation procedure by itself or from ingestion of tissue cysts from undercooked meat or from oocysts/sporozoites acquired from environmental source (11–13).

In the present work, it was performed experiments to verify the possibility of *T. gondii* transmission through BMT by using an experimental murine model. Our results demonstrated that it is possible to acquire toxoplasmosis due to BMT, when the bone marrow cells came from a donor seropositive for *T. gondii*. The experiments were carried out using two well-characterized strains of *T. gondii*, the RH (type I-high virulent strain) and ME-49 (type II-medium virulent strain). To determine the possibility of transmission during acute phase of infection animals were infected with RH-RFP and, after 3, 5, and 7 days of infection, bone marrow from these animals were harvested, when it was possible to confirm the *T. gondii* presence by both microscopy and qPCR analysis, suggesting a rapid migration of parasite to bone marrow. On the other hand, to evaluate if the parasite could persist in bone marrow even during latent chronic infection, bone marrow from mouse donors were infected with ME-49GFP and BM samples were collected on day 3, 5, 7, 15, and 30 after infection. The presence of *T. gondii* was also confirmed by microscopy and qPCR analysis, indicating that the parasite is able to reach BM during acute phase and remains there during chronic phase.

It is important to emphasize that, even though we aimed in the present study to observe the persistence of *T. gondii* in bone marrow, even during chronic phase of infection and allowing to assess the probability of parasite transmission after transplantation, it was also possible to observe the presence of extracellular parasites in the bone marrow cell preparations utilized for transplant procedure. However, we do not believe that the presence of extracellular tachyzoites in bone marrow could invalidate our hypothesis, as the presence of tachyzoite reinforces the premise that *T. gondii* reached the bone marrow and stayed there for the time required to achieve the chronic phase of infection. Indeed, at same time that it was possible to observe the presence of extracellular parasites, it was also possible to identify the presence of parasites inside of bone marrow cells, demonstrating the possibility of *T. gondii* transmission by these infected cells.

Even though it was not possible to observe in the present study the formation of *T. gondii* cysts in bone marrow cells, it was already described in the literature the presence of this parasite evolutive form, but only in bone marrow derived macrophages (23, 24). Thus, it is necessary to make clear that the findings described previously by those authors were only possible because, first of all, the bone marrow cells cultured *in vitro* were treated with supernatant from L929 fibroblasts, a conditioned medium as a source of MCSF (macrophage colony-stimulating factor). Also, the process of *T. gondii* cyst formation in macrophages differentiated from bone marrow cells, when cultured *in vitro*, was possible only because the macrophages were treated with IFN-γ, a key cytokine to induce cyst formation of this parasite. In addition, it has been also described other protocols to induce *T. gondii* cyst formation *in vitro*, as by CO_2_ starvation of the tissue culture, or by using medium with high a pH, or by temperature stress of the tissue culture at 43°C, instead of treatment of host cells by IFN-γ (23–25).

To verify the probability of *T. gondii* to infect bone marrow cells, *in vitro* experiments were carried out and it was observed that parasite is able to infect hematopoietic and nonhematopoietic BM cells. The presence of *T. gondii* on BM was predictable, as previous studies have indicated that this parasite is able to infect stem cells as well as resident BM macrophages, at least *in vitro* (26, 27). In the present study, the parasite burden was assessed by *in vivo* experiments and showed an increasing parasite load along the time of infection, indicating a parasite replication *in situ*, or a continuous migration to bone marrow. Interestingly, although parasite load during acute phase of infection was lower, the animals transplanted with bone marrow from donors infected with RH strain succumbed to infection faster than animals that received bone marrow from donors infected with ME49. This finding can be explained by the differential degrees of virulence between RH and ME49 strains. In fact, it is well-known that type I strains, as RH, are highly virulent to mice, whereas type II strains, as ME49, as well as type III strains, as VEG, present significant lower degree of virulence, but being able to produce tissue cysts (28). It has been described in the literature that seronegative recipients that receives BM cells from donors seropositive and chronically infected present no risk to acquire *T. gondii* infection (26, 29, 30). Our results, however, demonstrated that 100% of animals seroconverted after transplant from bone marrow cells from donors chronically infected by *T. gondii*.

In conclusion, our experimental study demonstrated that transmission of *T. gondii* through BMT from seropositive donors, at different time points of infection, and by strains with different degrees of virulence is an event that brings risk to the *T. gondii* seronegative recipients. Therefore, we are emphasizing that serological screening for both donors and recipients, in addition to *T. gondii* genomic DNA detection in bone marrow cells from donors before transplantation, are necessary procedures to avoid the risk of *T. gondii* infection, particularly because the allo-BM recipients are immunocompromised patients.

## Data Availability Statement

All datasets generated for this study are included in the manuscript/supplementary files.

## Ethics Statement

Ethical approval was obtained from Experimental Animals Committee of Federal University Uberlândia (Protocol # 109/16). All of the experimental procedures involving animals complied with 3 R principles according to the procedures established by the Universities Federation for Animal Welfare.

## Author Contributions

CL and JM designed the study. CL, TS, JA, and LC collected and analyzed data. CL performed statistical analysis and wrote the paper. JM and TM supervised the study and critically reviewed the manuscript.

### Conflict of Interest

The authors declare that the research was conducted in the absence of any commercial or financial relationships that could be construed as a potential conflict of interest.
